# Mutation Prevalence of Cerebral Cavernous Malformation Genes in Spanish Patients

**DOI:** 10.1371/journal.pone.0086286

**Published:** 2014-01-23

**Authors:** Rufino Mondéjar, Francisca Solano, Rocío Rubio, Mercedes Delgado, Ángel Pérez-Sempere, Antonio González-Meneses, Teresa Vendrell, Guillermo Izquierdo, Amalia Martinez-Mir, Miguel Lucas

**Affiliations:** 1 Servicio de Biología Molecular, Hospital Universitario Virgen Macarena, Facultad de Medicina, Sevilla, Spain; 2 Servicio de Neurología, Hospital Universitario Virgen Macarena, Facultad de Medicina, Sevilla, Spain; 3 Servicio de Neurología, Hospital Universitario de Alicante, Alicante, Spain; 4 Unidad de Dismorfología, Hospital Universitario Virgen del Rocío, Sevilla, Spain; 5 Unidad de Genética, Hospital Universitario Vall d'Hebron, Barcelona, Spain; 6 Instituto de Biomedicina de Sevilla (IBiS)/Hospital Universitario Virgen del Rocío/CSIC/Universidad de Sevilla, Sevilla, Spain; Instituto de Ciencia de Materiales de Madrid - Instituto de Biomedicina de Valencia, Spain

## Abstract

**Objective:**

To study the molecular genetic and clinical features of cerebral cavernous malformations (CCM) in a cohort of Spanish patients.

**Methods:**

We analyzed the *CCM1*, *CCM2*, and *CCM3* genes by MLPA and direct sequencing of exons and intronic boundaries in 94 familial forms and 41 sporadic cases of CCM patients of Spanish extraction. When available, RNA studies were performed seeking for alternative or cryptic splicing.

**Results:**

A total of 26 pathogenic mutations, 22 of which predict truncated proteins, were identified in 29 familial forms and in three sporadic cases. The repertoire includes six novel non-sense and frameshift mutations in *CCM1* and *CCM3*. We also found four missense mutations, one of them located at the third NPXY motif of CCM1 and another one that leads to cryptic splicing of *CCM1* exon 6. We found four genomic deletions with the loss of the whole *CCM2* gene in one patient and a partial loss of *CCM1*and *CCM2* genes in three other patients. Four families had mutations in *CCM3*. The results include a high frequency of intronic variants, although most of them localize out of consensus splicing sequences. The main symptoms associated to clinical debut consisted of cerebral haemorrhage, migraines and epileptic seizures. The rare co-occurrence of CCM with Noonan and Chiari syndromes and delayed menarche is reported.

**Conclusions:**

Analysis of CCM genes by sequencing and MLPA has detected mutations in almost 35% of a Spanish cohort (36% of familial cases and 10% of sporadic patients). The results include 13 new mutations of CCM genes and the main clinical symptoms that deserves consideration in molecular diagnosis and genetic counselling of cerebral cavernous malformations.

## Introduction

Cerebral Cavernous Malformations (CCMs; OMIM 116860) are enlarged vascular cavities without intervening brain parenchyma with an estimated prevalence in the general population close to 0.1–0.5 percent. Single or multiple malformations may develop, which can lead to cerebral haemorrhage (30–40%), seizures (40–70%), headache (10–30%) and focal neurological symptoms (35–50%). The onset age is variable with higher incidence between 10 and 40 years. CCM may occur sporadically or with an autosomal dominant inheritance pattern with variable expression and incomplete penetrance. Almost 25% of CCM carriers remain symptom-free throughout their lives.

Genes responsible for CCM were mapped [1] and located on 7q21.2 (*CCM1*, *KRIT1*) [2], [3], 7p13 (*CCM2*, *MGC4607*) [4] and 3q26.1 (*CCM3*, *PDCD10*) [5]. Close to 150 different mutations have been described in *CCM1* and almost all of them lead to premature stop codons that predict truncated proteins [6], [7]. Approximately 40 different mutations have been reported in the *CCM2* gene [4]. The encoded protein, malcavernin, contains a phosphotyrosine binding (PTB) domain, similar to the Krit1 binding partner ICAP1α. Krit1 interacts with Icap1α and malcavernin via their respective PTB domains [8]. Malcaverin has been described to interact with TrkA via its PTB domain to mediate TrkA-induced death in diverse cell types by a C-terminal “Karet” domain [9]. Finally, close to 15 different mutations have been described in the *CCM3* gene [5]. Recent findings describe the core complex formed by the association of the three proteins coded by CCM genes and their role in cytoskeletal remodelling, regulation of cell matrix adhesion and cell-cell junction homeostasis [10].

We have previously reported CCM mutations in *CCM1* and *CCM2* in patients of Spanish and Portuguese extraction. Here, we further studied *CCM1*, *CCM2* and *CCM3* in consecutive cases of Spanish patients including 94 CCM nuclear families and 41 sporadic cases. We report the identification of 13 novel mutations in the CCM genes, including the activation of a cryptic splicing signal. At the clinical level, we describe the main clinical symptoms of patients together with the rare coincidence of CCM with Chiari and Noonan syndromes and delayed menarche.

## Methods

### Patients

We recruited 94 consecutive families and 41 sporadic forms, which comprise 254 patients with clinical symptoms and gradient-echo MRI of CCM. Clinical assessment of patients included information on cerebral haemorrhage, epileptic seizures, headache and other neurological symptoms. The patients were classified as having hereditary or sporadic CCM on the basis of MRI and familial characteristics. Patients with at least one affected relative and/or multiple cavernous malformations in MRI were classified as having hereditary CCM. A written consent was obtained from the patients and their relatives included in this study. The study conformed to the tenets of the declaration of Helsinki and was approved by the “Committee of Ethics and Clinical Investigation” from Hospital Universitario Virgen Macarena.

### Molecular genetic analysis

Genomic DNA was extracted from peripheral blood using a salting-out standard procedure. The initial mutation search was performed by MLPA, using the SALSA P130 and P131 kits (MRC Holland) to detect genomic deletions in the CCM genes. Data analysis of MLPA was carried out with Coffalyser.NET Software (MRC Holland). If no deletions were found, coding and flanking intronic sequences were amplified with previously described primers for *CCM1* [2], [3] and *CCM2* [11]. Since *CCM1* has several variants, which differ in 5′UTR exons, the first coding exon was considered exon 1. We screened *CCM3* exons with a set of 7 pairs of primers (sequence of the primers available upon request). Sequencing of both the sense and antisense strands, extending 30–90 bases into the introns, was performed in a 3130 Genetic Analyzer (Applied Biosystems). When a non-sense mutation was identified we did not sequence the rest of genes. When a missense mutation was detected, we further sequenced the entire *CCM1*, *CCM2* and *CCM3* coding exons. When available, we performed blood RNA studies to evaluate alternative or cryptic splicing. Polyphen and SIFT prediction software were used to analyze missense mutations. Mutations were confirmed in an independent PCR amplification product either by sequencing of both forward and reverse strands or by restriction analysis. The mutations herein described were tested in approximately 200 chromosomes from healthy donors. In the case of gross deletions, we tried to delimit the breakpoints by reiterative PCR amplifications using primers extending up to approximately 2Kb 5′ and 3′ of the MLPA probes. In order to make data publicly available, mutations were submitted to the “Angioma Alliance” database (www.angiomaalliance.org, Durham, NC).

## Results

### *CCM1*

**Figure 1:**
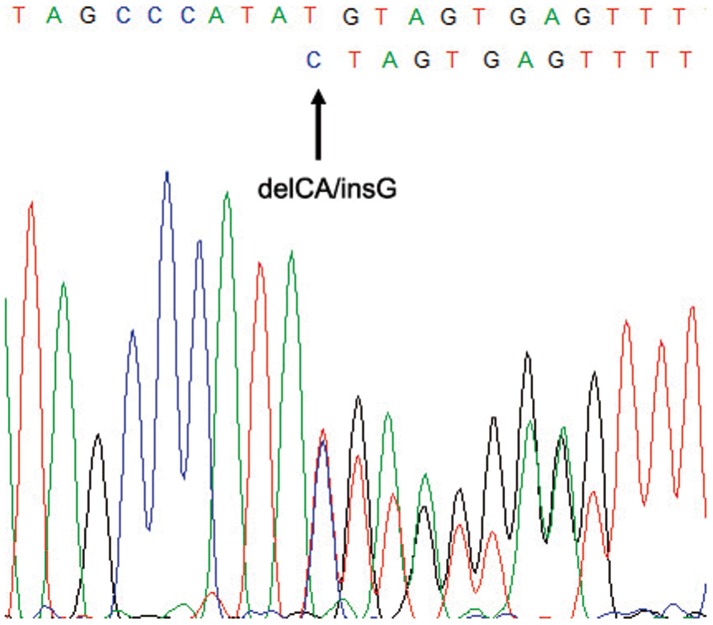
Sequencing of exon 5 (reverse strand, *CCM1*) of CV126 index patient showing mutation c.618_619delinsG.

**Figure 2:**
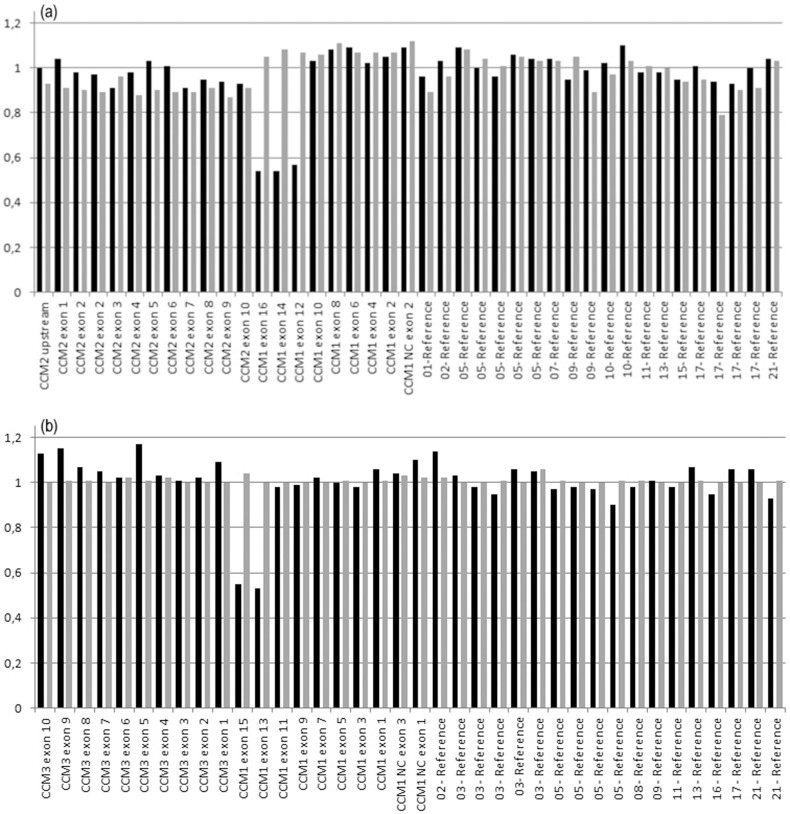
Quantitative MLPA analysis of (a) P130 and (b) P131 probemix (MRC Holland). Black columns represent CV146 index patient and grey columns represent a healthy control. The deletion of exons 12 to 16 of *CCM1* is shown.

Sequencing of coding exons and intronic boundaries of *CCM1* identified 20 mutations, eight of which were novel (table 1). A C-to-T transition at nucleotide 1114 resulted in nonsense mutation Q372X. Three frameshift mutations were identified: i) a nucleotide deletion in exon 6 (c.801delA, p.Lys267AsnfsX8); ii) an 11 bp deletion between positions 1314 to 1325 that predicts a truncated protein of 474 aminoacids; and iii) a small indel in exon 5 resulting in the deletion of CA at nucleotide position 618, and the insertion of one G, c.618_619delinsG (figure 1). Two gross genomic deletions were detected by MLPA in two unrelated patients, which encompass the three 5′non-coding exons and exons 12–16 (figure 2). Finally, we detected three nucleotide transitions at positions 691, 842 and 1775 that lead to the missense mutations p.N231D, p.D281G and p.S592T. None of these mutations had been previously reported (table 1). Known polymorphisms rs2027950 and rs11542682 were also detected.

**Table 1:** Mutations identified within the *CCM1* gene (NM_194455.1).

Pedigree	No. of affected individuals	Age at MRI	Exon/Intron^c^	Nucleotide change	Mutation consequence	Predicted amino acid change	Condition
CV171	6		NC 1–3	5′UTR	Gross deletion	NA [12]	Pathogenic
CV126	4	21–59	5	c.618_619delinsG^a^	Frameshift	p.His207ValfsX6	Pathogenic
CV122^b^	1		5	c.691A>G^a^	Missense	p.N231D	Possibly pathogenic
CV148	6	7–42	6	c.801delA^a^	Frameshift	p.Lys267AsnfsX8	Pathogenic
CV150^b^	1	69	6	c.842A>G^a^	Missense>altered splice site	p.Asp281GlyfsX5	Pathogenic
CV118	2	6, 40	7	c.880C>T	Nonsense	p.R294X [12]	Pathogenic
CVs 133, 163	3		7	c.902C>G	Nonsense	p.S301X [3]	Pathogenic
CV59	2	40,39	7	c.923T>A	Missense	p.L308H [14]	Unknown
CV87	7	9–61	7	c.968_971dupCACC	Frameshift	p.Ile325ThrfsX11 [14]	Pathogenic
CV160	2		8	c.1114C>T^a^	Nonsense	p.Q372X	Pathogenic
CV129	2		IVS9	c.1255-4delGTA	Altered splice site	NA [21]	Pathogenic
CV116	1	25	10	c.1314_1325del^a^	Frameshift	p.Gly439HisfsX36	Pathogenic
CV79	1	28	10	c.1360_1363delTCTC	Frameshift	p.Ser454LysfsX39 [6], [14]	Pathogenic
CV36	1	18	10	c.1362_1363delTC	Frameshift	p.Gln455ArgfsX23 [14]	Pathogenic
CV136^b^	1	8	12	c.1579G>A	Missense	p.A527T [6]	Pathogenic
CV105, 147	2	20, 43	IVS12	c.1730+5G>A	Altered splice site	NA [12]	Pathogenic
CV86	3	17–41	IVS12	c.1730+4delAGTA	Altered splice site	NA [6], [14]	Pathogenic
CV166	1	NA	13	c.1775G>C^a^	Missense	p.S592T	Unknown
CV10	5	4–80	14	c.1904InsA	Nonsense	p.Y635X [14]	Pathogenic
CV146	2	20	12–16	Exons 12–16 del^a^	Genomic deletion	NA	Pathogenic

a New mutation non-previously reported.

b Sporadic patient.

c Exon count begins with exon 1 as the first coding exon for *CCM1*.

NA: not avialable. NC: 5′ untranslated exons.

**Figure 3:**
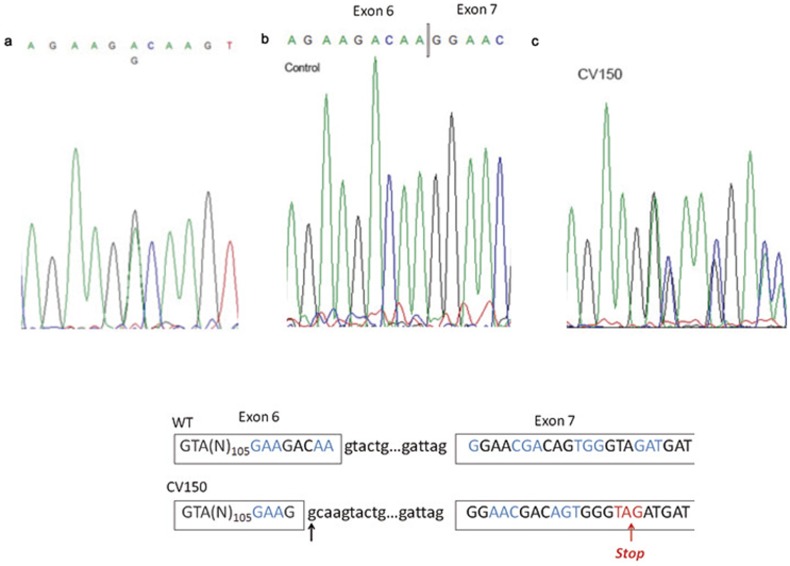
Mutation analysis in the CV150 patient. (a) Sequencing of exon 6 (*CCM1* gene), which shows the A>G transition at position 842. (b) cDNA sequencing of exons 5 to 8 of a healthy subject and (c) the CV150 patient. A new 5′ splice site is created at the mutation site. The new splicing alters the open reading frame of exon 7 and generates a premature stop codon (p.Asp281GlyfsX5). (d) Diagram showing the cryptic splicing of exon 6 to exon 7 in the patient. Codons are shown in consecutive blue and black colour and premature stop codon in red colour. The arrow shows the novel donor splice site.

We analysed two missense mutations at the cDNA level. Mutation c.842A>G in exon 6 of *CCM1* predicts the aminoacid change D281G. Nevertheless, sequencing of the patient's cDNA revealed that mutation D281G resulted in an altered donor site at the point of mutation (figure 3), which causes a cryptic splicing, alters the open reading frame and predicts a truncated protein of 286 aminoacids (p.Asp281GlyfsX5). On the other hand, missense mutation p.S592T did not modify the wild-type splicing.

### *CCM2*

The *CCM2* gene was studied in non-*CCM1* patients by MLPA and sequencing. MLPA detected two gross genomic deletions. One of them included the whole genomic sequence covered by the MLPA probes, while the second deletion affected the 5′UTR and the first exon (table 2). The size of these two deletions could not be accurately determined by successive PCRs up to 2 Kb around the MLPA probes. Sequencing of *CCM2* detected three previously described nonsense and frameshift mutations, c.55C>T (p.R19X), c.169_172delAGAC and c.554_567del (table 2). Two non-previously described point mutations were also identified: i) the nucleotide transversion 713C>A that leads to missense mutation p.S238Y; and ii) the silent G>A transition at position 222. Polymorphisms rs2107732 (exon 2), rs2304689 (intron 3), rs11552377 (exon 4), rs2289367 (exon 8) and rs190686229 (intron 9) were found in 21, 3, 12, 10 and 2 families, respectively.

**Table 2:** Mutations identified within the *CCM2* gene (NM_031443.3).

Pedigree	No. of affected individuals	Age at MRI	Exon/Intron	Nucleotide change	Mutation consequence	Predicted amino acid change	Condition
CV77	1	50	All	delCCM2	Gross deletion	NA [15]	Pathogenic
CV128	5	39–72	1	5′UTR-exon1del	Gross deletion	NA [12], [15]	Pathogenic
CV140	1	55	2	c.55C>T	Nonsense	p.R19X [22]	Pathogenic
CV100	1	41	2	c.169_172delAGAC	Frameshift	p.Arg57CysfsX1 [14]	Pathogenic
CV145	2		3	c.222G>A^a^	Transition	None	Non pathogenic
CV^b^	15	22–78	5	c.554_567del	Frameshift	p.Ala186GlyfsX44 [14]	Pathogenic
CV114	1		6	c.713C>A^a^	Missense	p.S238Y	Possibly pathogenic

a New mutation non-previously reported.

b The c.554_567del is a redundant mutation that was detected in 11 unrelated Spanish families [14].

NA: not available.

### *CCM3*

We studied *CCM3* by MLPA and sequencing in *CCM1*- and *CCM2*-negative patients. MLPA failed to detect deletions in the coding sequence in the patients. Sequencing of the *CCM3* gene uncovered three novel point mutations: i) a G-to-C transition at the splicing donor site of intron 7; ii) deletion c.211delA that predicts a truncated protein of 88 aminoacids; and iii) an A insertion at nucleotide 538 causing a frameshift followed by a stop codon at residue 183 (table 3). We also detected the intronic variant c.268+53C>T (intron 5).

**Table 3:** Mutations identified within the *CCM3* gene (NM_007217).

Pedigree	No. of affected individuals	Age at MRI	Exon/Intron	Nucleotide change	Mutation consequence	Predicted amino acid change	Condition
CV139	1		5	c.211delA^a^	Frameshift	p.Ser71AlafsX18	Pathogenic
CV127	1		IVS6	c.395+1G>C^a^	Altered splice site	NA	Pathogenic
CV164^b^	1		IVS7	c.474+5G>A	Altered splice site	p.Asp133HisfsX10 [17]	Pathogenic
CV125	2		8	c.538dupA^a^	Frameshift	p.Tyr180AsnfsX3	Pathogenic

a New mutation non-previously reported.

b Sporadic patient.

NA: not available.

### Clinical outcomes

The main clinical symptoms at debut of the disease were headaches (36%), epileptic seizures (25%), instability and dizziness (18%), migraines (12%), hemorrhage (5%), and myoclonic seizures (1%). Three cases were discovered after MRI in patients with clinical diagnosis of: i) Noonan syndrome associated to Arnold-Chiari malformation; ii) a nine year old girl with premature menarche; and iii) a patient with clinical diagnosis of possible metastasis secondary to non-cerebral cancer (table 4).

**Table 4:** Clinical description of patients with mutations detected in CCM genes.

Pedigree	Mutation	No. lesions	Size^a^	Location	Epileptic seizures	Hemorrhage	Headache	Other
CV126	CCM1: c.618_619delinsG	Multiple	3×4 cm	Front at bilateral, predominantly right at parietal, temporal head bilateral caudate nucleus, midbrain and pons.	Yes	Yes	No	Left paresthesia from 7 years
CV 148	CCM1: c.801delA	Multiple (17)	-	Supra and infratentorial, in both cerebral and cerebellar hemispheres. Mid-level in pontine brainstem.	-	-	-	
CV118	CCM1: c.880C>T	Multiple	-	-	-	-	-	Chiari and Noonan syndromes
CV 133	CCM1: c.902C>G	2	-	Pineal region and right temporal	-	-	-	
CV 163	CCM1: c.902C>G	Multiple (50–70)	-	Cerebral hemispheres, cerebellum and brainstem	-	Yes	-	
CV 116	CCM1: c.1314_1325del	Multiple	-	-	Yes	Yes	No	
CV136	CCM1: c.1579G>A	2		Frontal and parietal right side	-	-	Yes	Delayed menarche
CV 105	CCM1: c.1730+5G>A	Multiple	12×10 mm	Subcortical area of convolutions. Left side	No	Yes	No	
CV 147	CCM1: c.1730+5G>A	Multiple	4×3 cm	Occipital right lobe. Small lesions in supra and infratentorial level	No	Yes	Yes	
CV 136^b^	CCM1: c.1579G>A	1	-	Front region	-	-	Yes	
CV 146 II1	CCM1: Exon 12-16del	4	-	-	Yes	No	Yes	
CV 146 I1	CCM1: Exon 12-16del	2	-	-	No	No	Yes	
CV 114	CCM2: c.713C>A	Multiple	-	Cerebellar peduncle, supra and infratentorial and spinal cord.	No	Yes	No	
CV 125	CCM3: c.538dupA	Multiple	-	Both hemispheres	Yes	Yes	No	
CV 139	CCM3: c.214delA	Multiple	-	-	-	-	Yes	

a Size of the larger lesion.

b Sporadic patient.

## Discussion

Mutations in the *CCM1*, *CCM2* and *CCM3* genes account for 65%, 19% and 16% of CCM cases, respectively [12]. Here we describe the genetic study of a group of 94 and 41 cases of familial and sporadic CCM, comprising a total of 254 patients. We report the identification of 31 mutations in the CCM genes, 13 of which have not been previously described. The identified mutations include nonsense, frameshift, missense and splice variants. Interestingly, RNA studies have revealed the use of a cryptic splice site leading to a frameshift by one of the missense mutant alleles.

The prevalence of mutations in this study, 36% and 10% for familial and sporadic forms, respectively, is relatively lower than previous reports [12], [13]. Among the 31 mutations identified in this work, 13 of them are novel. It is worth mentioning that among the nearly 200 different mutations that have been described in CCM patients thus far, there is a very low degree of recurrence among different families. Two exceptions are the c.1363C>T transition in the *CCM1* gene, which is highly prevalent in the Hispanic-Mexican population [3] and a 14 bp-deletion in *CCM2* previously detected in patients of Spanish and Portuguese descent [14].

In this work, we identified a total of 31 mutations including 5 nonsense, 10 frameshift and 6 missense mutations and four exonic deletions. Using MLPA, we were able to detect four genomic deletions in four unrelated families. One of the patients carries a deletion encompassing the whole *CCM2* gene, as previously reported [15]. The deletion of three non-coding exons of *CCM1*in patient CV171 leads to loss of the transcription initiation site and therefore absence of the full-length transcript should be expected. The deletion of *CCM1* exons 12–16 was identified in a single nuclear family. Finally, we identified one deletion that encompasses the ATG start codon of *CCM2* and that expands into the 5′UTR. The breakpoints of these deletions could not be accurately determined using a long-range PCR apporach. Therefore, they were defined relative to the position of the MLPA-DNA probes.

The nature of *CCM1* mutations has been reported to be highly stereotyped leading to premature termination codon through different mechanisms, including missense mutations that activate cryptic splice sites [6], [7], [12]. In this line, we were able to analyze an RNA sample of two patients with missense mutations p.D281G and p.S592T in the *CCM1* gene. Mutation p.D281G generates a novel donor site leading to cryptic splicing (figure 3), which alters the open reading frame and predicts a truncated protein of 286 aminoacids (p.Asp281GlyfsX5). On the other hand, mutation p.S592T in *CCM1* showed both alleles at cDNA level and no cryptic splicing event was found. The mutated residue is located in the F2 FERM domain of Krit1. At the protein level serine 592 is conserved among different species and is predicted to be damaging according Polyphen (score: 0.709) and tolerated by SIFT (score: 0.08). Therefore, the pathogenicity of the p.S592T mutation remains unclear. Although cDNA analysis of the p.N231D mutation in *CCM1* could not be carried out, Polyphen (score: 0.982) and SIFT (score: 0) analysis point out to a pathogenic mutation. Finally, among the polymorphisms found in the *CCM1* gene, a silent substitution at nt 1980 (rs11542682) was detected in a heterozygous state in 12% in our patients, a slightly lower frequency than previously reported [6]. The frequency of the intronic transition c.989+63C>G (rs2027950) was similar to other populations.

The analysis of *CCM2* in our cohort revealed two new mutations. RNA study of CV145 index patient, carrier of the silent transition 222G>A, showed both mutant and wild type alleles and did not reveal aberrant splicing of *CCM2*. Therefore, the transition should be considered as avariant without pathogenic significance [12]. While RNA was not available to study the c.713C>A (p.S238Y) mutation, bioinformatic analysis suggests that this mutation would be pathogenic (Polyphen score: 0.993; SIFT score: 0.03). Moreover, p.S238 is a phosphorylation site [16] and the mutation could affect to *CCM2* function. The frequency of polymorphisms c.157G>A (rs2107732), c.205-36A>G (rs2304689), c.358G>A (rs11552377), c.915G>A (rs2289367) and c.1054+12C>T (rs190686229) were similar to other populations and to the 1000 genomes project database (http://www.1000genomes.org/ensembl-browser; accessed 10 Apr 2013). Some of these polymorphisms had been associated with a significant increase in the risk for CCM and a predisposition to a higher occurrence of a ‘potentially disabling’ symptomatology rather than to a ‘possibly life-threatening’ symptomatology [13]. In addition we studied some previously described polymorphisms which are very frequent in our patients' cohort: c.157G>A, c.358G>A and c.915G>A. None of them showed an altered cDNA sequence and no evidence of alternative splicing was found.

The mutation responsible for the disease remains undiscovered in 65% of familial forms in this work. Several explanations have been proposed for this apparent low detection: i) the existence of mosaicism or genomic deletions non-detected by the exon-by-exon sequencing approach [5]; ii) the existence of an additional fourth *CCM locus* close to *CCM3* [17]; and iii) deep intronic mutations that could activate alternative or cryptic sites [18]. In both, patients and controls, several aberrant splicing events in *CCM1* gene have been described in peripheral blood mononuclear cells [19]. We aimed to analyze this possibility in our cohort of patients and controls. Preliminary results indicated the lack of association of the detected isoforms with the disease (data not shown).

Regarding the clinical symptoms in the patients' cohort, we studied four unrelated families with radiological images of developmental venous anomalies (DVAs) and CCMs. None of them carried a pathogenic mutation in the CCM genes. Moreover, the father and the daughter of CV146 carry of a gross deletion in *CCM1*, whereas the son, who has radiological images of DVA, did not carry the deletion. In the light of a recent report [20], our findings support a separate pathogenic mechanism for DVAs and CCMs. Besides the classical clinical features of CCM, the main findings in the patients studied here are the coincidence of cavernomas with Chiari and Noonan syndromes and with delayed menarche. It is worth to point out that these casual findings do not imply a pathogenic association between cavernomas and the above-described syndromes.

We can conclude that the analysis of CCM genes by sequencing and MLPA has detected mutations in almost 35% of a Spanish cohort (36% of familial cases and 10% of sporadic patients). The results comprise 13 non-previously described mutations, including the aminoacid change p.D281G that leads to cryptic splicing and thus, a frameshift mutation. Moreover, the co-occurrence of additional syndromes together with CCM deserves consideration in molecular diagnosis and genetic counseling of cerebral cavernous malformations.
